# Non-Motor Symptoms in Patients with Parkinson’s Disease – Correlations with Inflammatory Cytokines in Serum

**DOI:** 10.1371/journal.pone.0047387

**Published:** 2012-10-17

**Authors:** Daniel Lindqvist, Eli Kaufman, Lena Brundin, Sara Hall, Yulia Surova, Oskar Hansson

**Affiliations:** 1 Department of Clinical Sciences, Section for Psychiatry, Lund University, Lund, Sweden; 2 Psychiatry Skåne, Lund, Sweden; 3 Department of Translational Science and Molecular Medicine, Michigan State University, Grand Rapids, Michigan, United States of America; 4 Department of Clinical Sciences, Lund University, Lund, Sweden; 5 Department of Neurology, Skåne University Hospital, Lund, Sweden; 6 Clinical Memory Research Unit, Department of Clinical Sciences, Lund University, Malmö, Sweden; Philadelphia VA Medical Center, United States of America

## Abstract

**Background:**

Parkinson’s Disease (PD) is the second most common neurodegenerative disorder of the central nervous system. Motor symptoms are the focus of pharmacotherapy, yet non-motor features of the disease (e.g. fatigue, mood disturbances, sleep disturbances and symptoms of anxiety) are both common and disabling for the patient. The pathophysiological mechanisms behind the non-motor symptoms in PD are yet to be untangled. The main objective of this study was to investigate associations between pro-inflammatory substances and non-motor symptoms in patients with PD.

**Methods and Materials:**

We measured C-reactive protein, interleukin (IL)-6, soluble IL-2 receptor (sIL-2R) and tumor necrosis factor-α (TNF-α) in blood samples from PD patients (n = 86) and healthy controls (n = 40). Symptoms of fatigue, depression, anxiety and sleeping difficulties were assessed using the Functional Assessment of Chronic Illness Therapy-Fatigue (FACIT), the Hospital Anxiety and Depression Scale (HAD), and the Scales for Outcome in PD-Sleep Scale respectively.

**Results:**

IL-6 was significantly higher in PD patients than in healthy controls. Compared to healthy controls, PD patients displayed significantly higher mean scores on HAD and lower scores on FACIT, thus indicating more severe symptoms as measured with these scales. Within the PD sample, high levels of both sIL-2R and TNF-α were significantly associated with more severe symptoms assessed by means of FACIT and HAD (depression and anxiety subscales). SIL-2-R levels were able to significantly predict FACIT and HAD scores after the effects of age, gender, anti-parkinsonian medications, and severity of motor symptoms were controlled for.

**Discussion:**

We suggest that non-motor symptoms in PD patients, such as fatigue and depressive symptoms, might be generated via inflammatory mechanisms. This knowledge might contribute to the development of novel treatment options in PD, specifically targeting non-motor symptoms.

## Introduction

Parkinson’s Disease (PD) is a chronic, neurodegenerative disease affecting approximately 2% of the population over the age of 60 and 4% of those over the age of 80 [1]. In addition to the vast personal suffering PD causes patients and relatives, the disease also imposes significant direct costs on public services [2]. Although most research has focused on the motor symptoms of PD, several studies show that non-motor aspects of the disease, such as depressive symptoms, fatigue, sleep impairment and apathy, are very common and significantly predicts disability and the patients self-reported quality of life (QoL) [3]–[6]. The prevalence of depressive symptoms is higher in PD than in other chronic degenerative disorders [7], and studies show that such symptoms may precede the motor symptoms in PD [8].

Several hypotheses have been postulated regarding possible shared pathophysiologic mechanisms between the core pathophysiology of PD and the depressive symptoms in PD patients. “The inflammatory hypothesis” is based on the notion that inflammatory mechanisms might be involved in the pathophysiology of PD [9] as well as Major Depressive Disorder (MDD) [10]. PD patients show signs of peripheral and central inflammation, including elevated cytokines in serum [11] and cerebrospinal fluid (CSF) [12], as well as activated microglia [13]. Peripheral blood monocytes isolated from PD patients produce larger amounts of several cytokines, including tumor necrosis factor alpha (TNF-α), than healthy controls - indicating that the elevated serum levels of cytokines are symptoms of immunological dysregulation, rather than just secondary to the dopaminergic cell degeneration [14]. Some of these signs are also demonstrable in depressed, non-PD patients. For example, several studies report elevated cytokines such as interleukin-6 (IL-6) and TNF-α as well as soluble interleukin-2 receptor (sIL-2R) in serum [15] of MDD patients compared with controls. Interestingly, Pålhagen and colleagues reported a neurobiological distinction between patients with PD and MDD and patients with solely MDD, in that the latter group displayed higher levels of corticosterone and IL-6 in CSF [16]. In a recent review by Barnum & Tansey, it was suggested that inflammation might contribute to the development of non-motor PD symptoms [17]. Only a few clinical studies have, however, investigated potential associations between such symptoms and peripheral cytokines. Menza et al. showed that TNF-α in serum is correlated with several non-motor symptoms, including cognition and depressive symptoms [18], and Scalzo et al showed that IL-6 correlated with scores on the Mini-Mental State Examination (MMSE) in PD patients without dementia [11].

As studies on inflammatory markers and non-motor aspects of PD are scarce, we wanted to further explore this area. In this study we measured four pro-inflammatory substances in the blood of 86 PD-patients and 40 controls, evaluated for non-motor symptoms such as fatigue, depression, anxiety, and sleeping difficulties. We wanted to compare the groups for cytokine levels and symptoms severity, and finally investigate correlations between cytokines and non-motor symptoms. We report significant differences in IL-6 levels and severity of non-motor symptoms between PD patients and controls. Symptoms of fatigue, depression, and anxiety were associated with cytokines in serum.

## Materials and Methods

### Ethics Statement

The Ethics Committee of Lund University approved this study. All study participants gave written consent for participation in the study, which was performed in accordance with the provisions of the Helsinki Declaration.

### Study Participants

Eighty-six PD patients and 40 healthy controls were enrolled in the study between 2008 and 2012. All patients were recruited from different neurological clinics in southern Sweden, and were invited to the neurological clinic at Skåne University Hospital, Lund for enrollment in the study. In many cases, the healthy controls were spouses of patients, or otherwise part of their extended family. A thorough medical history was taken and a routine laboratory screen was carried out. Exclusion criteria were a diagnosis of dementia, acute or chronic inflammatory disease and ongoing treatment with NSAIDs or corticosteroids. In addition, one patient was excluded from the study due to widespread malignancy at the time of clinical assessment and blood sampling. The exclusion criteria were applied to both patients and controls. During the visit, a licensed and experienced medical doctor evaluated the subject using the Unified Parkinson’s Disease Rating Scale (UPDRS), and verified the PD diagnosis according to the NINDS Diagnostic Criteria [19]. Demographic characteristics of patients and controls are given in Table 1.

**Table 1:** Demographic characteristics of patients and controls.

	Controls (n = 40)	Patients (n = 86)	Sign
Sex	f = 26 (65%), m = 14	f = 35 (41%), m = 51	.01^a^
Age (yrs, mean ± SD)	64.8±9.0	64.2±10.8	.75^c^
Illness duration (yrs, mean ± SD)		6.9±6.2	
Hoehn and Yahr *(mean ± SD)*		1.9±0.9	
Schwab & England *(mean ± SD)*	99.8±1.6	90.9±8.0	.00^c^
UPDRS motor score *(mean ± SD)*	1.5±2.6	17.3±10.4	.00^c^
CRP (median, IQL)	1.3, .6–2.4	1.1, .6–2.5	.58^b^
IL-6 (median, IQL)	2.8, 2.8–2.8	2.8, 2.8–4.0	.02^b^
sIL-2R (median, IQL)	399.0, 332.0–543.0 *(n = 39)*	425.0, 332.3–539.5	.52^b^
TNF-α (median, IQL)	9.5, 7.0–11.8	10.0, 8.0–12.0	.10^b^
HAD-anxiety (median, IQL)	1.0, .0–6.0 *(n = 38)*	3.5, 2.0–6.0 *(n = 84)*	.00^b^
HAD-depression *(median, IQL)*	.0, .0–2.0 *(n = 39)*	2.5, 1.0–5.0 *(n = 84)*	.00^b^
FACIT-fatigue *(median, IQL)*	51.0, 49.0–52.0 *(n = 36)*	43.0, 36.0–48.0 *(n = 79)*	.00^b^
SCOPA-sleep night *(mean ± SD)*	3.4±2.7 *(n = 39)*	4.4±3.2 *(n = 84)*	.13^c^
Asthma/allergies, n (%)	5 (13)	10 (12)	.89^a^
Cardiovascular disease, n (%)	13 (33)	18 (21)	.16^a^
Diabetes mellitus, n (%)	1 (3)	3 (3)	.77^a^
Osteoarthritis, n (%)	4 (10)	5 (6)	.40^a^

a Pearson’s chi-square.

b Mann-Whitney.

c Student’s t-test.

### Evaluation of Non-motor Symptoms

The study participants were asked to complete the following surveys during their visit: Functional Assessment of Chronic Illness Therapy-Fatigue (FACIT) [20], Hospital Anxiety and Depression Scale (HAD) (including subscales for symptoms of depression as well as anxiety) [21], and Scales for Outcomes in PD-Sleep (SCOPA-S) [22]. Complete HAD anxiety scores were available from 84 patients and 38 controls, complete HAD depression scores from 84 patients and 39 complete FACIT scores from 79 patients and 36 controls, and complete SCOPA-S scores from 84 patients and 39 controls.

### Blood Sampling and Biological Assays

In the morning and immediately after the clinical evaluations, serum samples were drawn into 5 ml test tubes and analyzed immediately at the Department of Clinical Immunology at the Skåne University Hospital in Lund. Chemiluminescent assays (Immulite 1000 Siemens) were used for all analyses. Inter-assay variation is consistently less than 10% and this assay is quality optimized for routine clinical serum cytokine analysis at Lund University Hospital. Monoclonal antibodies specific for the immunological biomarker tested were coated on a solid phase (polystyren beads). Serum samples were incubated with the solid phase antibody for 30 minutes, and thereafter a polyclonal anti-antibody, labeled with alkaline phosphatase, was added and the samples were incubated for another 30 minutes. Unbound conjugate was washed away and chemiluminescent substrate added, and following another ten minutes, a luminometer was used to measure the light emission from the chemical reaction. A concentration was then calculated using samples with known concentration as references. The detection limits were 2.8 ng/L for IL-6, 1.7 ng/L for TNF-α, 5 kU/L for sIL-2R and 0.6 mg/L for C-reactive protein (CRP). In the entire sample, 84 (66.7%) of the subjects had IL-6 levels below the detection limit of 2.8 ng/L, and were therefore assigned the value 2.8. In the same manner, 40 (31.7%) of the subjects had CRP levels below the detection limit of 0.6 mg/L, and were subsequently assigned the value 0.6. None of the other cytokines were outside of detection range.

### Statistical Analyses

The Statistical Package for the Social Sciences (SPSS) for Mac was used for statistical calculations. CRP, IL-6, sIL-2R and TNF-α were all non-normally distributed, hence the Mann-Whitney U-test was used for group-wise comparisons and Spearman’s rho for correlative analyses. Pearson’s chi-squared test was used to compare proportions. In order to control for the effect of gender and age, one-way analysis of covariance (ANCOVA) were conducted as described below. To further explore significant correlations between cytokines and symptoms, hierarchical multiple regressions were carried out as also described below. After exclusion of two subjects with extreme values on FACIT and HAD depression, all variables entered in the regression models were normally distributed. Hierarchical multiple regressions were carried out both with and without the outliers, and results from both analyses are given in the results section. P-values below 0.05 were considered significant.

## Results

### Demographics

Overall demographics, including the most common somatic co-morbidities, of the two groups are given in Table 1. Mean disease duration in the patient group was 4.7 years. The groups did not differ significantly in age. The gender distribution, however, differed significantly between the two groups (Pearson’s c^2^ = 6.46, p<.01), with a higher proportion of men in the patient group. This observation thus mandates special attention to the gender variable as a potential confounder in subsequent group comparisons. Status as a *de novo* PD patient or the use of antidepressant medications had no significant impact on any of the measured cytokines (data not shown). Twenty-two PD patients reported dyskinesias at the time of the blood sampling. These patients did not differ significantly in any of the measured cytokines compared to the ones who did not report dyskinesias (N = 64) (Mann-Whitney U tests, all p-values>0.24).

### Non-motor Symptoms in in Patients and Controls

Compared to controls, patients with PD displayed significantly higher mean scores on the HAD depression subscale (Mann-Whitney U = 1080.0, p<.001), the HAD anxiety subscale (Mann-Whitney U = 921.0, p<.01) as well as significantly lower scores on FACIT-fatigue (Mann-Whitney U = 451.5, p<.001), thus indicating more severe symptoms in the PD group. The SCOPA-sleep night scale did not differ significantly between the two groups (Student’s t = 1.45, p>.15, ns). In the entire sample there were no significant gender differences in mean scores on FACIT or the HAD subscales (Mann-Whitney U tests, all p-values >0.14), thus making gender unlikely as a confounder in these analyses. Women had, however, significantly higher mean scores on SCOPA-S compared to men (Mann-Whitney U = 1485.0, p = .04). Subsequent ANCOVA with SCOPA-S as the dependent variable and gender and diagnose as fixed factors did not reveal any significant effect of patient/control on SCOPA-S scores when the effect of gender was controlled for (p>.05, ns).

### CRP, IL-6, sIL-2R and TNF-α in Patients and Controls

Mean or median absolute values of CRP, IL-6, sIL-2R and TNF-α of patients and controls are given in Table 1. We found that IL-6 was significantly higher in patients than in healthy controls (Mann-Whitney U = 1344.5, p = .02). In contrast, there were no significant differences in the levels of CRP, sIL-2R or TNF-α between the two groups. When exploring gender differences, mean scores of IL-6 and CRP did not differ significantly between males and females. There were, however, significant gender differences in levels of sIL-2R (Mann-Whitney U = 1499.0, p = .03) and TNF-α (Mann-Whitney U = 1466.0, p = .01) – thus making gender a potential confounder in these two comparisons. ANCOVAS were therefore carried out as described in the section “dealing with potential confounders”.

As previously noted, some two thirds of the study participants had IL-6 levels below the detection limit of 2.8 ng/L. In order to ensure that this did not skew our results, we also performed a Chi-square test for independence with Yates Continuity Correction on the IL-6 scores divided into detectable (n = 41) or undetectable (n = 85). The result indicated a significant association between detectable IL-6 and PD status (Pearson’s c^2^ = 5.08, p<.01).

### Correlations between Inflammatory Markers and Non-motor Symptoms

In the patient group, absolute values of TNF-α correlated significantly with FACIT scores (Spearman’s Rho = −0.19, p = .05), HAD depression scores (Spearman’s Rho = 0.35, p = .001) as well as the HAD anxiety scores (Spearman’s Rho = 0.21, p = .03). Further on, sIL-2R correlated significantly with FACIT scores (Spearman’s Rho = −0.34, p = .001), HAD depression scores (Spearman’s Rho = 0.38, p = .001) as well as the HAD anxiety scores (Spearman’s Rho = 0.24, p = .01). High scores on HAD equals more severe symptoms of depression and anxiety while low scores of FACIT reflect more severe fatigue, hence all the significant correlations above shows that high levels of cytokines are associated with more severe symptomatology. There were no significant correlations between any of the non-motor symptoms scales and IL-6 or CRP. SCOPA-S scores did not correlate significantly with any inflammatory biomarker. In the control group, there were no significant correlations between cytokines and symptom scores.

### Dealing with Potential Confounders

Since there were significant gender differences in sIL-2R and TNF-α (as opposed to the other variables analyzed in the group wise comparisons), we conducted two separate ANCOVAS with the natural logarithms of sIL-2R and TNF-α as dependent variables respectively. Male/female and patient/control were entered as fixed factors and age as a covariate in each model. After adjusting for the effect of gender and age, there were no significant differences between the two groups in TNF-α (F (1, 122) = 2.07, p = .15, ns) or sIL-2R (F (1, 119) = 0.007, p = .93, ns) levels.

Since there was a trend for a greater proportion of individual with cardiovascular disease in the control group, we performed an additional analysis, excluding all patients and controls with cardiovascular disease and repeated the group comparisons (68 PD patients & 27 healthy controls). The difference in IL-6 levels remained significant (Mann-Whitney U = 705.5, p = 0.04) and in addition PD patients displayed significantly higher levels of TNF-alpha (Mann-Whitney U = 674.5, p = 0.04).

In order to control for potential confounders in the correlative analyses, we conducted three hierarchical regression models with FACIT (one outlier excluded) (Table 2), HAD depression (two outliers excluded) (Table 3), and HAD anxiety scores (Table 4) entered as dependent variable in each respective model. Age, gender UPDRS motor score, and levodopa dose equivalents [23] for each patient were entered into the first block, and the natural logarithms of TNF-α and sIL-2R were additionally entered into the second block. After controlling for confounders, sIL-2R (but not TNF-α) significantly predicted FACIT scores (β = −0.31, p = .014), HAD depression scores (β = 0.36, p<.003), and HAD anxiety scores (β = 0.32, p<.008). UPDRS motor score significantly predicted FACIT scores (β = −0.23, p = .0046). Levodopa dose equivalents were not significantly associated with any of the non-motor symptoms, nor cytokine levels (Spearmańs Rho, all p-values >.12).

**Table 2:** Hierarchical regression with FACIT as dependent variable. Standardized β-coefficients.

	Block 1	Block 2
Sex	−.008	.10
Age	.04	.18
UPDRS motor score	−.25*	−.24*
Levdodopa-equivalent	−.15	−.14
TNF-α		−.10
sIL-2R		−.31*
R^2^	.09	.19*

* p<.05.

**Table 3:** Hierarchical regression with HAD depression as dependent variable. Standardized β-coefficients.

	Block 1	Block 2
Sex	.06	−.008
Age	.08	−.07
UPDRS motor score	.19	.15
Levodopa-equivalent	.15	.16
TNF-α		.13
sIL-2R		.37**
R^2^	.08	.23**

** p<.01.

**Table 4:** Hierarchical regression with HAD anxiety as dependent variable. Standardized β-coefficients.

	Block 1	Block 2
Sex	−.22	−.34**
Age	−.19	−.34**
UPDRS motor score	.20	.16
Levodopa-equivalent	−.01	−.22
TNF-α		.13
sIL-2R		.33**
R^2^	.07	.19**

** p<.01.

The procedures were repeated with the outliers included. The main results were similar, with the exception that TNF-α and UPDRS motor scores also became significant in the second model with HAD depression as dependent variable (β = 0.31, p<.005; β = 0.28, p<.009).

## Discussion

### Main Findings

We measured four different pro-inflammatory substances in blood samples of 86 PD patients and 40 controls, all assessed for symptoms of depression, anxiety, fatigue, and sleeping difficulties. Our most salient findings were that the PD patients had more severe non-motor symptoms and displayed significantly higher levels of IL-6 than the controls. Moreover, both sIL-2R and TNF-α correlated significantly and positively with symptoms of depression, anxiety, and fatigue. In the subsequent hierarchical regression analyses, we confirmed that sIL-2R levels had a unique and significant contribution in explaining the variance of FACIT, HAD depression and anxiety scores, and this association remained significant even after controlling for the potentially confounding effects of age, gender, motor symptom severity, and anti-parkinsonian medication.

### PD Patients Display Higher Levels of IL-6 than Controls

PD patients had significantly higher mean levels of IL-6 than controls, and a significantly greater proportion of the PD patients displayed IL-6 above the detection limit compared to controls. These findings are replications of earlier studies showing that PD patients have increased IL-6 levels in serum [11], [24] and CSF [25]. Whether these elevations are specific for PD has not yet been determined. IL-6 is an important cytokine in the innate immune response, and has been found to be elevated in such diverse conditions as suicidal depression [26] and heart disease [27]. Also within the group of movement disorders, the specificity of IL-6 elevations has been questioned. In a study by Brodacki et al., patients with atypical PD (n = 7) displayed the highest elevations of IL-6, with mean vales significantly different from patients with idiopathic PD. We did not include patients with atypical PD in the present study, but this would be of interest in the future.

### Symptoms of Depression and Anxiety are More Pronounced in PD Patients and Related to Signs of Inflammation

We report that PD patients display significantly more severe symptoms of depression and anxiety than healthy controls. This adds to the vast body of previous research suggesting that these kinds of symptoms are more common in patients with PD than in the general population [28]. Interestingly, we found associations between symptom severity and TNF-α and sIL-2R levels. Depression scores were in general more strongly associated with pro-inflammatory cytokines than were anxiety scores, as evident from the bivariate correlative analyses. In the subsequent regression analyses we showed that sIL-2R (and less robustly TNF-α) explained a unique and substantial part of the variance in depression scores, and that this relationship was statistically significant. Our findings are mainly in line with a study by Menza and colleagues in which a significant association between plasma TNF-α and depressive symptoms was found in a group of PD patients with ongoing depression [18]. SIL-2R was not, however, measured in this study.

To the best of our knowledge, this is the very first study to investigate associations between cytokines and symptoms of anxiety in PD patients. Even in non-PD samples, potential immune-related pathophysiological mechanisms behind symptoms of anxiety are less well characterized than for depressive symptoms. Recently, Hou & Baldwin addressed this issue in a review where they concluded that findings have been inconsistent across studies [29]. Hence, our finding that the level of anxious symptoms is associated with pro-inflammatory cytokines is the first of its kind, and future replications are warranted.

### Fatigue in PD – a Specific Inflammatory Symptom?

To the best of our knowledge, this is the first time an association between fatigue and pro-inflammatory cytokines in PD patients has been investigated. We report a significant correlation between sIL-2R and severity of fatigue, even when the potentially confounding effects of age, gender, motor symptom severity and anti-parkinsonian medications were controlled for. Even though fatigue is a common and disabling symptom in PD it often goes undetected, has an unknown cause and specific therapies are lacking. Hagell and Brundin showed in 2009 that symptoms of anxiety and depression, rather than e.g. sleep quality or daytime sleepiness, could predict fatigue in PD patients [30]. The combination of symptoms such as fatigue, depressed mood, social withdrawal and pain in conjunction with infections is referred to as “sickness behavior”, which is thought to be generated via pro-inflammatory cytokines acting on the brain [31]. Since elevated cytokine levels have been found also in presumed non-infectious diseases, such as PD and MDD, it may be suggested that cytokines play a role in generating the prominent “sickness-behavior”-associated symptoms often seen in these disorders. Interestingly, a robustly designed study by Raison and colleagues showed that also milder forms of fatigue (as opposed to chronic fatigue) is associated with increased inflammation, even after adjusting for depressive status [32]. Our finding that degree of fatigue, as measured by FACIT, significantly correlated with sIL-2R, strengthens this hypothesis and adds to the ever-growing pile of reports suggesting that a chronically active immune response plays a key role in the pathophysiology of fatigue, irrespective of diagnosis.

### The Role of sIL-2R in the Generation of Non-motor Symptoms in PD – a Novel Finding

The inflammatory biomarker most robustly associated with non-motor symptoms was sIL-2R. We did, however, not find any significant differences between patients and controls. We are aware of only one earlier study that has investigated sIL-2R in PD patients [33]. Non-motor symptoms were not investigated in this study, but in line with our results, no difference in sIL-2R between patients and controls was found. This soluble form of the IL-2 receptor is predominantly produced through enzymatic cleavage of the membrane-bound IL-2 receptor in T-cells [34]. IL-2 is one of the major cytokines, and has a key role in the activation of the adaptive immune response. When a T-helper cell is presented with its antigen on the MHC class II molecule of an antigen-presenting cell, the T-cell becomes activated and starts to multiply by producing both IL-2 and its membrane-bound receptor. Through this receptor, IL-2 acts as an autocrine and paracrine mitogenic factor, thereby driving the monoclonal expansion [35]. While it is theoretically possible that the correlation between fatigue and sIL-2R levels observed in our material results only from increased cleavage of membrane-bound IL-2 receptor in patients with more severe fatigue, the consensus seems to be that there is a strong correlation between sIL-2R and actual IL-2 levels [34] and thus putative T-cell activation [36].

In our bivariate correlational analyses, sIL-2R consistently covaried to a higher degree with the outcome measures (non-motor symptoms) than did TNF-α. In the regression models sIL-2R consistently contributed significantly to explaining the variance in the dependent variable while TNF-α did not. The only exception for this was the regression model with HAD depression scores as the dependent variable and two outliers included. In this model TNF-α also reached significance. It thus appears as much of the ability of TNF-α to predict the outcome scores stems from its shared variance with sIL-2R. One can only speculate why sIL-2R showed the strongest relationship with non-motor symptoms. Different physiological roles of the measured cytokines may be one explanation. Another may be that the values of CRP, IL-6 (and to a lesser extent TNF-α) tended to be below or close to the detection limit while sIL-2R levels displayed a larger spread.

### Can Anti-inflammatory Drugs Treat Non-motor Symptoms in PD?

Our results support the notion that inflammatory mechanisms may contribute to the development of PD pathology in general and non-motor symptoms in specific. The potential usefulness of anti-inflammatory drugs in the prevention and treatment of PD has been explored in previous studies. Some epidemiologic reports have suggested that Non-Steroid-Anti-Inflammatory-Drugs (NSAID) confer protection against the development of PD [37], and some experimental studies have suggested that NSAIDs can attenuate dopamine depletion in the striatum of rats [38], [39]. A recent Cochrane-review, however, did not find robust evidence for recommending NSAIDs as primary or secondary prevention of PD [40]. Depressive symptoms in PD patients can be difficult to treat with conventional antidepressants [41]. We are not aware of any previous studies where anti-inflammatory drugs have been used to treat non-motor symptoms of PD. However, there are preliminary clinical trials of MDD patients without PD indicating that NSAIDs as add-on to conventional antidepressants may have beneficial effects on depressive symptoms [42], [43]. Based on our results and data from these studies, it would be interesting to further explore whether anti-inflammatory drugs are effective in treating non-motor symptoms in PD patients.

### No Association between Cytokines and Levodopa-induced Dyskinesias

Results from some previous animal studies have suggested a link between neuroinflammation and levodopa-induced dyskinesias [44]. We did not, however, find any significant differences in cytokine blood levels between patients who suffered from dyskinesias and those who did not. One of the reasons for the negative findings might be that we had a relatively large number of samples below the detection limit. Since the putative connection between dyskinesias and neuroinflammation is highly interesting, we aim to explore this in future cerebrospinal fluid studies using high-sensitivity assays.

### Conclusions

To summarize, our results show that PD patients display significantly higher levels of IL-6, but not CRP, sIL-2R or TNF-α, compared to healthy controls. PD patients also reported more pronounced fatigue, depression and anxiety, but not increased sleeping difficulties, on self-assessment scales. We found significant correlations between fatigue, depression and anxiety on one hand, and TNF-α and sIL-2R, but not CRP or IL-6, on the other hand. When further investigated with hierarchical regressions, sIL-2R but not TNF-α contributed significantly to explaining the variance in non-motor symptom scores. Our findings, together with some earlier studies, build a strong case that pro-inflammatory cytokines may play a role in generating non-motor symptoms in PD. Hopefully, this will eventually lead to the development of new treatment strategies based on anti-inflammatory mechanisms.

## References

[1] de Lau LM, Breteler MM (2006) Epidemiology of Parkinson’s disease. Lancet Neurol 5: 525–535. 16713924 10.1016/S1474-4422(06)70471-9

[2] Findley L, Aujla M, Bain PG, Baker M, Beech C, et al (2003) Direct economic impact of Parkinson’s disease: a research survey in the United Kingdom. Mov Disord 18: 1139–1145. 14534917 10.1002/mds.10507

[3] Soh S-E, Morris ME, McGinley JL (2011) Determinants of health-related quality of life in Parkinson’s disease: A systematic review. Parkinsonism Relat Disord 17: 1–9. 20833572 10.1016/j.parkreldis.2010.08.012

[4] McKinlay A, Grace RC, Dalrymple-Alford JC, Anderson T, Fink J, et al (2008) A profile of neuropsychiatric problems and their relationship to quality of life for Parkinson’s disease patients without dementia. Parkinsonism Relat Disord 14: 37–42. 17627863 10.1016/j.parkreldis.2007.05.009

[5] Weintraub D, Moberg PJ, Duda JE, Katz IR, Stern MB (2004) Effect of psychiatric and other nonmotor symptoms on disability in Parkinson’s disease. J Am Geriatr Soc 52: 784–788. 15086662 10.1111/j.1532-5415.2004.52219.x

[6] Menza M, Dobkin RD, Marin H, Bienfait K (2010) Sleep disturbances in Parkinson’s disease. Mov Disord 25 Suppl 1S117–122. 20187236 10.1002/mds.22788 PMC2840057

[7] Nilsson FM, Kessing LV, Sørensen TM, Andersen PK, Bolwig TG (2002) Major depressive disorder in Parkinson’s disease: a register-based study. Acta Psychiatrica Scandinavica 106: 202–211. 12197858 10.1034/j.1600-0447.2002.02229.x

[8] Jacob EL, Gatto NM, Thompson A, Bordelon Y, Ritz B (2010) Occurrence of depression and anxiety prior to Parkinson’s disease. Parkinsonism Relat Disord 16: 576–581. 20674460 10.1016/j.parkreldis.2010.06.014 PMC2963655

[9] Barnum CJ, Tansey MG (2010) Modeling neuroinflammatory pathogenesis of Parkinson’s disease. Prog Brain Res 184: 113–132. 20887872 10.1016/S0079-6123(10)84006-3

[10] Schiepers OJ, Wichers MC, Maes M (2005) Cytokines and major depression. Prog Neuropsychopharmacol Biol Psychiatry 29: 201–217. 15694227 10.1016/j.pnpbp.2004.11.003

[11] Scalzo P, Kummer A, Cardoso F, Teixeira AL (2010) Serum levels of interleukin-6 are elevated in patients with Parkinson’s disease and correlate with physical performance. Neuroscience Letters 468: 56–58. 19857551 10.1016/j.neulet.2009.10.062

[12] Blum-Degen D, Müller T, Kuhn W, Gerlach M, Przuntek H, et al (1995) Interleukin-1 beta and interleukin-6 are elevated in the cerebrospinal fluid of Alzheimer’s and de novo Parkinson’s disease patients. Neuroscience Letters 202: 17–20. 8787820 10.1016/0304-3940(95)12192-7

[13] Ouchi Y, Yagi S, Yokokura M, Sakamoto M (2009) Neuroinflammation in the living brain of Parkinson’s disease. Parkinsonism and realted Disorders 15: S200–S204. 10.1016/S1353-8020(09)70814-4 20082990

[14] Reale M, Iarlori C, Thomas A, Gambi D, Perfetti B, et al (2009) Peripheral cytokines profile in Parkinson’s disease. Brain, Behavior, and Immunity 23: 55–63. 10.1016/j.bbi.2008.07.003 18678243

[15] Liu Y, Ho RC-M, Mak A (2011) Interleukin (IL)-6, tumour necrosis factor alpha (TNF-α) and soluble interleukin-2 receptors (sIL-2R) are elevated in patients with major depressive disorder: A meta-analysis and meta-regression. Journal of Affective Disorders: 1–10. 10.1016/j.jad.2011.08.003 21872339

[16] Pålhagen S, Qi H, Mårtensson B, Wålinder J, Granérus A-K, et al (2009) Monoamines, BDNF, IL-6 and corticosterone in CSF in patients with Parkinson’s disease and major depression. Journal of Neurology 257: 524–532. 19844754 10.1007/s00415-009-5353-6

[17] Barnum CJ, Tansey MG (2012) Neuroinflammation and Non-motor Symptoms: The Dark Passenger of Parkinson’s Disease? Curr Neurol Neurosci Rep. In Press. 10.1007/s11910-012-0283-6 22580742

[18] Menza M, Dobkin RD, Marin H, Mark MH, Gara M, et al (2010) The Role of Inflammatory Cytokines in Cognition and Other Non-Motor Symptoms of Parkinson’s Disease. Psychosomatics 51: 474–479. 21051678 10.1176/appi.psy.51.6.474 PMC2987579

[19] Gelb DJ, Oliver E, Gilman S (1999) Diagnostic criteria for Parkinson disease. Arch Neurol 56: 33–39. 9923759 10.1001/archneur.56.1.33

[20] Cella D (1997) Manual of the Functional Assessment of Chronic Illness Therapy (FACIT) Measurement System. Evanston IL: Center on Outcomes, Research and Education (CORE), Evanston Northwestern Healthcare and Northwestern University.

[21] Zigmond AS, Snaith RP (1983) The hospital anxiety and depression scale. Acta Psychiatr Scand 67: 361–370. 6880820 10.1111/j.1600-0447.1983.tb09716.x

[22] Marinus J, Visser M, van Hilten JJ, Lammers GJ, Stiggelbout AM (2003) Assessment of sleep and sleepiness in Parkinson disease. Sleep 26: 1049–1054. 14746389 10.1093/sleep/26.8.1049

[23] Tomlinson CL, Stowe R, Patel S, Rick C, Gray R, et al (2010) Systematic review of levodopa dose equivalency reporting in Parkinson’s disease. Mov Disord 25: 2649–2653. 21069833 10.1002/mds.23429

[24] Dobbs RJ, Charlett A, Purkiss AG, Dobbs SM, Weller C, et al (1999) Association of circulating TNF-alpha and IL-6 with ageing and parkinsonism. Acta Neurol Scand 100: 34–41. 10416510 10.1111/j.1600-0404.1999.tb00721.x

[25] Muller T, Blum-Degen D, Przuntek H, Kuhn W (1998) Interleukin-6 levels in cerebrospinal fluid inversely correlate to severity of Parkinson’s disease. Acta Neurol Scand 98: 142–144. 9724016 10.1111/j.1600-0404.1998.tb01736.x

[26] Lindqvist D, Janelidze S, Hagell P, Erhardt S, Samuelsson M, et al (2009) Interleukin-6 is elevated in the cerebrospinal fluid of suicide attempters and related to symptom severity. Biol Psychiatry 66: 287–292. 19268915 10.1016/j.biopsych.2009.01.030

[27] Dinh W, Futh R, Nickl W, Krahn T, Ellinghaus P, et al (2009) Elevated plasma levels of TNF-alpha and interleukin-6 in patients with diastolic dysfunction and glucose metabolism disorders. Cardiovasc Diabetol 8: 58. 19909503 10.1186/1475-2840-8-58 PMC2778641

[28] Aarsland D, Marsh L, Schrag A (2009) Neuropsychiatric symptoms in Parkinson’s disease. Mov Disord 24: 2175–2186. 19768724 10.1002/mds.22589 PMC2787875

[29] Hou R, Baldwin DS (2012) A neuroimmunological perspective on anxiety disorders. Hum Psychopharmacol 27: 6–14. 22213434 10.1002/hup.1259

[30] Hagell P, Brundin L (2009) Towards an understanding of fatigue in Parkinson disease. J Neurol Neurosurg Psychiatry 80: 489–492. 19204024 10.1136/jnnp.2008.159772

[31] Dantzer R, Kelley KW (2007) Twenty years of research on cytokine-induced sickness behavior. Brain Behav Immun 21: 153–160. 17088043 10.1016/j.bbi.2006.09.006 PMC1850954

[32] Raison CL, Lin JM, Reeves WC (2009) Association of peripheral inflammatory markers with chronic fatigue in a population-based sample. Brain Behav Immun 23: 327–337. 19111923 10.1016/j.bbi.2008.11.005

[33] Kluter H, Vieregge P, Stolze H, Kirchner H (1995) Defective production of interleukin-2 in patients with idiopathic Parkinson’s disease. J Neurol Sci 133: 134–139. 8583216 10.1016/0022-510x(95)00180-a

[34] Caruso C, Candore G, Cigna D, Colucci AT, Modica MA (1993) Biological significance of soluble IL-2 receptor. Mediators Inflamm 2: 3–21. 18475497 10.1155/S0962935193000018 PMC2365387

[35] Smith KA (1988) Interleukin-2: inception, impact, and implications. Science 240: 1169–1176. 3131876 10.1126/science.3131876

[36] Rubin LA, Nelson DL (1990) The soluble interleukin-2 receptor: biology, function, and clinical application. Ann Intern Med 113: 619–627. 2205142 10.7326/0003-4819-113-8-619

[37] Gagne JJ, Power MC (2010) Anti-inflammatory drugs and risk of Parkinson disease: a meta-analysis. Neurology 74: 995–1002. 20308684 10.1212/WNL.0b013e3181d5a4a3 PMC2848103

[38] Sairam K, Saravanan KS, Banerjee R, Mohanakumar KP (2003) Non-steroidal anti-inflammatory drug sodium salicylate, but not diclofenac or celecoxib, protects against 1-methyl-4-phenyl pyridinium-induced dopaminergic neurotoxicity in rats. Brain Res 966: 245–252. 12618347 10.1016/s0006-8993(02)04174-4

[39] Teismann P, Ferger B (2001) Inhibition of the cyclooxygenase isoenzymes COX-1 and COX-2 provide neuroprotection in the MPTP-mouse model of Parkinson’s disease. Synapse 39: 167–174. 11180504 10.1002/1098-2396(200102)39:2<167::AID-SYN8>3.0.CO;2-U

[40] Rees K, Stowe R, Patel S, Ives N, Breen K, et al. (2011) Non-steroidal anti-inflammatory drugs as disease-modifying agents for Parkinson’s disease: evidence from observational studies. Cochrane Database Syst Rev: CD008454. 10.1002/14651858.CD008454.pub2 PMC13579550 22071848

[41] Weintraub D, Morales KH, Moberg PJ, Bilker WB, Balderston C, et al (2005) Antidepressant studies in Parkinson’s disease: a review and meta-analysis. Mov Disord 20: 1161–1169. 15954137 10.1002/mds.20555 PMC1989731

[42] Muller N, Schwarz MJ, Dehning S, Douhe A, Cerovecki A, et al (2006) The cyclooxygenase-2 inhibitor celecoxib has therapeutic effects in major depression: results of a double-blind, randomized, placebo controlled, add-on pilot study to reboxetine. Mol Psychiatry 11: 680–684. 16491133 10.1038/sj.mp.4001805

[43] Akhondzadeh S, Jafari S, Raisi F, Nasehi AA, Ghoreishi A, et al (2009) Clinical trial of adjunctive celecoxib treatment in patients with major depression: a double blind and placebo controlled trial. Depress Anxiety 26: 607–611. 19496103 10.1002/da.20589

[44] Barnum CJ, Eskow KL, Dupre K, Blandino P Jr, Deak T, et al (2008) Exogenous corticosterone reduces L-DOPA induced dyskinesia in the hemi-parkinsonian rat: role for interleukin1-beta. Neuroscience 156: 30–41. 18687386 10.1016/j.neuroscience.2008.07.016 PMC2615135

